# Numerical Simulation of the Deformation Behavior of Softwood Tracheids for the Calculation of the Mechanical Properties of Wood–Polymer Composites

**DOI:** 10.3390/polym14132574

**Published:** 2022-06-24

**Authors:** Robert Hartmann, Florian Puch

**Affiliations:** 1 Plastics Technology Group, Faculty of Mechanical Engineering, Technische Universität Ilmenau, 98683 Ilmenau, Germany; florian.puch@tu-ilmenau.de; 2 Thüringisches Institut für Textil- und Kunststoff-Forschung e.V., 07407 Rudolstadt, Germany

**Keywords:** pine, densified wood, softwood tracheid, wood polymer composite, multi-scale model, representative volume element (RVE)

## Abstract

From a fiber composite point of view, an elongated softwood particle is a composite consisting of several thousand tracheids, which can be described as fiber wound hollow profiles. By knowing their deformation behavior, the deformation behavior of the wood particle can be described. Therefore, a numerical approach for RVE- and FEM-based modelling of the radial and tangential compression behavior of pine wood tracheids under room climate environment is presented and validated with optical and laser-optical image analysis as well as tensile and compression tests on pine sapwood veneer strips. According to the findings, at 23 °C and 12% moisture content, at least 10 MPa must be applied for maximum compaction of the earlywood tracheids. The latewood tracheids can withstand at least 100 MPa compression pressure and would deform elastically at this load by about 20%. The developed model can be adapted for other wood species and climatic conditions by adjusting the mechanical properties of the base materials of the cell wall single layers (cellulose, hemicellulose, lignin), the dimensions and the structure of the vessel elements, respectively.

## 1. Introduction

### 1.1. Research Motivation

With an annual production volume of more than 12 million m^3^, Germany is one of the most important manufacturers of panel-shaped wood-based materials in Europe [1,2]. These materials are currently particularly in demand due to their CO_2_ storage capability (one cubic meter of wood binds one ton of CO_2_), suitability for substituting non-renewable raw materials and low costs (especially softwood) [3,4,5]. To meet the constantly growing requirements for efficient lightweight constructions in transport, logistics, construction and furniture applications, current research focuses on environmentally friendly binder systems, efficient processing processes and new modeling approaches for property assessment, which leads to an improvement in the achievable material properties from wood-based materials. Wagenführ states that nature-optimized material wood can only be used in the best conceivable way with comprehensive knowledge of the anatomical structure, the chemical composition, the physical properties and the processing conditions [6]. Wagenführ also describes how new types of hybrid composites with outstanding material properties can be produced through particle decomposition, press treatment and hybridization of wood, and emphasizes that it is advantageous to preserve the cell structure of the wood as far as possible and to view the wood cell microscopically as a fiber composite material [7]. To implement this recommendation, this publication uses numerical simulation methods for filament wound hollow profiles to model the compaction deformation behavior of tracheids to estimate achievable mechanical properties of highly densified, elongated softwood particles such as veneer strips and OSB strands, which are already used successfully to manufacture wood–polymer composites (WPC) [8,9].

### 1.2. Material Potential and Structural Composition of Softwood

**Figure 1:**
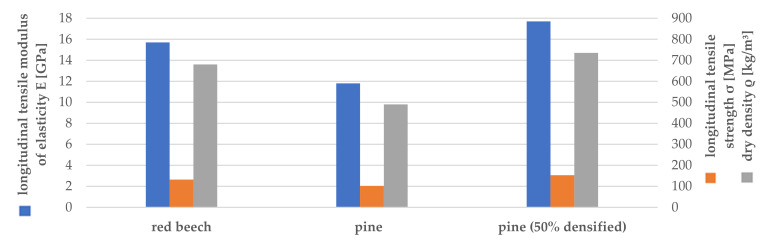
Average tensile modulus of elasticity, tensile strength and density for solid wood of red beech and pine as well as a hypothetical estimate for 50% compressed pine wood.

To exploit the material potential of softwood, its structural composition must be considered during processing. This includes, on the one hand, utilizing the excellent mechanical properties in the growth, respectively, longitudinal direction and, on the other hand, the specific compaction of the porous softwood structure while retaining the strength-giving components [10]. Figure 1 shows a comparison of the material properties of red beech and pine [11] as well as the hypothetical achievable properties of pine at 50% compression. It clearly shows that pine wood can at least match or even surpass the properties of beech wood through the targeted compaction.

Moon et al. describe the softwood structure on eight scaling levels: trunk (1 m), horizontal trunk cross-section (1 cm), annual growth rings (1 mm), wood cell (500 µm), tracheid (25 µm), multilayer tracheid cell wall (300 nm), microfibril as a composite of elementary fibrils (10 nm) and the cellulose molecule (1 nm) as a composite of glucose or cellobiose units [12]. The cellulose molecules as the main component of softwood can have an amorphous or crystalline structure and are linked in a fibrous manner. The elementary fibril is made up of approx. 50 cellulose fibers arranged in parallel, which are shifted by one glucose molecule to form hydrogen bonds. The amorphous cellulosic structures make the elementary fibrils flexible and allow them to be stretched lengthwise. The microfibrils are surrounded by fibrous hemicelluloses and embedded in lignin. Microfibrils, hemicelluloses and lignin form the cell wall structure of tracheids, which are formed as a rectangular hollow profile in the form of four individual layers (ML/P—middle lamella and primary wall, S1—secondary wall layer 1, S2—secondary wall layer 2, S3—secondary wall layer 3), which differ in layer thickness, fiber content and orientation [13,14,15,16]. These tracheid cells with a diameter of approx. 25 to 50 µm and a length of approx. 0.5 to 5 mm form the approx. 500 µm large wood cells arranged in annual rings as early (EW) and late wood (LW) tracheids [17]. According to Mahlke, EW tracheids are thin-walled, have a large lumen and are used to transport water and nutrients. Due to their chisel-shaped flat ends, the cells are connected to one another in a butt joint-like manner. LW tracheids, on the other hand, are thick-walled and have narrow lumens and are used for mechanical strength and rigidity [18]. Their ends interlock like wedges, which significantly increase the transmittable force and the achievable strength compared to butt joints. EW and LW tracheids also differ significantly in their cell wall structure (alignment of the microfibrils, layer thickness, number, shape of the bordered pits, etc.) [19]. According to Hasenstab, this explains the difference in density between EW (0.25 g/cm^3^) and LW (0.75 g/cm^3^) [20]. The growth rings as well as the sapwood and heartwood areas, are visible in the cylindrical horizontal cross-section of the trunk. According to Wagenführ and Scholz, the younger outer growth rings are called sapwood. This is where water and nutrient transport takes place for some growing seasons. With increasing age, the sapwood loses vitality and turns into heartwood. Heartwood contains colored, mostly phenolic substances that impregnate the cell walls and increase the durability of the wood. The tree interrupts the connections between the cells by closing the bordered pits so that capillary exchange is no longer possible. Hence, heartwood is mechanically reinforced by the stored substances and protected against microbial destruction. When heated, the stored substances escape from the wood in the liquid or gaseous form [21]. The basic building blocks cellulose, hemicellulose and lignin react very differently to changes in temperature and humidity. Furuta et al. describe that the matrix polymer lignin is hydrophobic and exhibits a glass transition temperature range (i.e., starts to soften) of about 50 °C to 100 °C. The hemicellulose and cellulose fibers, on the other hand, are hygroscopic and have a glass transition temperature of approx. 40 °C or over 100 °C in [22]. The decomposition of the cellulose fibers begins at around 180 °C. Temperatures of around 170 °C must be regarded as the upper limit for processing because pyrolytic degradation processes then set in. During these processes, hydrophilic OH groups between hemicellulose and lignin are converted, leading to physical changes in the wood structure, which degrade the mechanical properties.

### 1.3. Process Dependent Mechanical Properties of Softwood

As a result, the elasticity of the cell wall depends significantly on the prevailing processing conditions and decreases, according to Wolcott, from approx. 22 GPa at 8 wt% moisture and 30 °C to approx. 13.5 GPa at 8 wt% moisture and 100 °C and 8 GPa at a residual moisture content of almost 0 wt% and 225 °C [23]. This change in the elasticity of the cell wall has a significant influence on the mechanical properties and the deformation behavior of the used wood particles. Wagenführ and Scholz demonstrate that the longitudinal modulus of elasticity of softwood depends on the “fiber” load angle, moisture content, temperature, density and load duration [21]. The mechanical strength and elasticity of the softwood increase quasi-linearly with its density, i.e., with the ratio of EW to LW tracheids. The ratio decreases with the width of the growth rings. Schoenfelder et al. show that the percentage of LW at 1 mm ring width is about 55% and at 2 mm ring about 42% [24]. The modulus of elasticity decreases significantly with an increasing difference in the angle between the direction of growth and the load direction (corresponds to the fiber-load angle known from fiber composite technology), increasing humidity and temperature [21]. In the case of increasing or decreasing moisture content below the fiber saturation range (approx. 30% for softwood), the direction-dependent swelling and shrinkage behavior of the wood particles must be considered. For pine wood, the shrinkage or swelling is 0.36% tangentially, 0.19% radially and 0.01% longitudinally for every 1% change in moisture content [25]. Hence, the wood particles will vary primarily in their width and height during processing during thermal compression above the boiling point of water. The following hypothesis can be derived from the research: By numerical modeling of the deformation behavior of the wood cell as a combination of EW and LW tracheids, the optimal process parameters can be derived, which enable targeted compression of the softwood, in which the compaction of EW tracheids is maximized, and the mechanically stable LW tracheids are retained, thus minimizing the cross-sectional area with the same bearable tensile force resulting in increased tensile strength and stiffness of the wood particle.

### 1.4. Modelling Transverse Deformation Behavior of Wood

To characterize the large deformation behavior of the tracheid microstructure of spruce wood under axial and transverse compression loading, Zhong et al. developed a numerical simulation model based on the representative volume element (RVE) method and showed that the main failure modes for transverse compression are cell wall folding and collapse [26]. Yan et al. presented a 3D FEM model for transverse compression of the microstructure of chemically treated wood using ABAQUS/Explicit software. In addition to the good correlation of numerical predictions with microscopic images of compacted wood, the calculated stress–strain curve for lateral compression also resembles the experimental findings found in the relevant research [27]. Wang et al. developed a hierarchical ABAQUS FEM model for the softwood structure based on EW and LW cells to analyze stress concentrations and predict the initial fracture of the wood cell walls under different loads such as longitudinal-radial or longitudinal-tangential in-plane shear loading and radial or longitudinal tensile stress. The simulated regions of stress concentration of the tracheid wall matched the measured initial fracture locations well. The initial fracture position of the cell occurs under in-plane shear loading at the S1–S2 interface and under longitudinal tensile stress at the edges of the S2 layer [28]. De Magistris et al. presented an ABAQUS FEM model for simulating the deformation of wet wood under transverse compression and combined transverse shear and compression to analyze the mechanical pulping of wood chips in refiners. A network of 12 cells was used to replicate the structure of wood, and it was concluded that the deformation behavior was determined by the cell structure itself. The difference in material properties when using an isotropic single layer or an orthotropic multilayer structure only slightly affected the deformation pattern. Therefore, it is of great relevance to depict the anatomical peculiarities of the cell structure as precisely as possible [29]. Narin demonstrates that the material point method (MPM) can be used to model large-scale, morphology-based structures such as wood. He used a micrograph of wood and discretized it into an MPM model. After discretization, the MPM calculations were very stable when calculating the large deformations of wood during transverse compression using elasto-plastic properties and automatically modeled contacts between cell walls [30].

In this paper, an extended approach is presented to model the EW and LW tracheids with particular attention to their anatomical features as fiber wound composite hollow profiles and to use these models for an RVE- and FEM-based simulation to examine their radial and tangential compression behavior.

## 2. Materials and Methods

### 2.1. Simulation of Compression Behavior of Tracheids

As a first step, RVE homogenization was conducted to calculate the mechanical properties of the cell wall layers for a wood moisture content of 12% at room temperature. Instead of the 8.3% present in the test specimens (see chapter materials), 12% moisture content was simulated since most of the mechanical values of the basic building blocks of the cell wall are available in the research for this degree of moisture. Subsequently, the material parameters of the unidirectional layers were exported as a material data sheet and used to construct the cell wall of EW and LW tracheids in Ansys Composite PrepPost. The temperature- and moisture-dependent modification of the mechanical properties of cellulose, hemicellulose and lignin has not yet been represented in the RVE model and is to be implemented as part of further research work by using the relevant findings known from research [31,32,33,34,35] or determined by experiments. Then, the built models were used to simulate the deformation behavior of 0.1 mm long representative cutouts of EW and LW tracheids during compression perpendicular to their longitudinal axis (radially as well as tangentially) to determine the level of compression pressure at which the EW tracheids fail and the LW tracheids deform purely elastically.

#### 2.1.1. Simulative Estimation of Mechanical Properties of Cell Wall Single Layers

Based on the findings of [15,36,37,38,39,40,41,42], the mechanical properties of the basic building blocks of the cell wall single layers were selected, as shown in Table 1.

Table 2 displays the selected dry mass fractions, thicknesses and microfibril angles for EW and LW tracheids [13,14,15].

**Figure 2:**
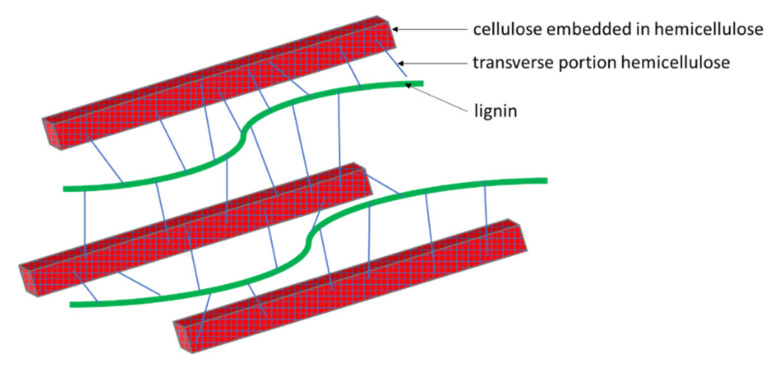
Schematic representation of the microstructure of lignocellulosic plant cell walls (hemicellulose—blue, cellulose—red, lignin—green) [43].

The following simplifications were made when creating the RVE model: no transverse portion of hemicellulose was modeled (according to research, about 15% of the respective dry mass fraction), lignin was modeled as an isotropic matrix instead of anisotropic fibers and no trapped air pores were taken into account (Figure 2).

The dry mass fractions shown in Table 2 were used to calculate the hemicellulose, cellulose and lignin fractions at 12 wt% moisture. These moisture-adjusted mass fractions were then used together with the values from Table 1 to calculate the mechanical properties of the cell wall single layers by using the Software MSC Digimat, Version 2021.2, MSC Software GmbH, Garching, Germany (Table 3). The modelling results show good agreement with the research values [44].

#### 2.1.2. Digital Representation of Tracheid Structures

**Figure 3:**
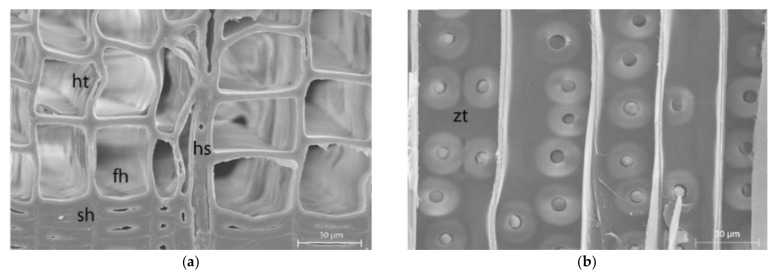
SEM images of softwood in cross-section (fh earlywood, sh latewood, ht bordered pits, hs ray) (**a**) and in radial section (zt twin bordered pits) (**b**) Reprinted with permission from Ref. [45]. 2012, Rosenthal.

The average dimensions of EW and LW tracheids were assumed according to the findings of Rosenthal and Bäucker, Sallet et al. and Havimo et al. (Figure 3) [45,46,47].

**Figure 4:**
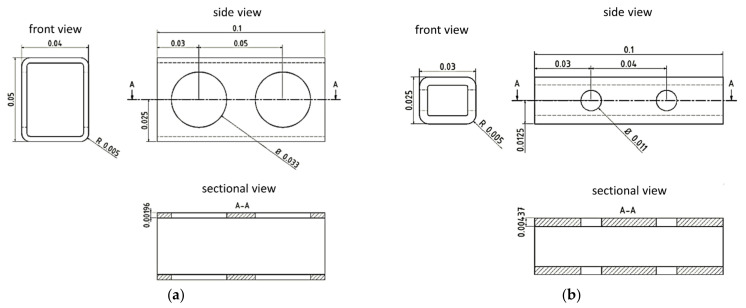
Dimensions in mm of EW (**a**) and LW (**b**) tracheid CAD model.

A representative length of 0.1 mm was selected and according to Qu et al., the membrane diameter of the bordered pits was estimated at about 70% of the radial lumen height (Figure 4) [48]. Because both types of tracheids show bordered pits in the tangential cell walls (LW less than EW), it is to be expected that their radial compressive strength will be lower than their tangential compressive strength.

To estimate the density of the tracheids, their cross-sectional areas of cell wall and lumen were set in relation to each other. The results (EW 0.251 g/cm^3^ and LW 0.802 g/cm^3^) correlate with other research [20]. Table 4 displays the laminate setup chosen to replicate the single layers of the tracheid cell wall based on the research values cited above (Table 2). A quasi-isotropic layer structure with 0/45/−45/90° orientation was selected for the ML/P layer, and the layer thickness was quartered accordingly. The S1 layer was constructed in a two-ply structure with a winding angle of 60 and −60°. The S2 layer was built up for EW at 25° and for LW at a 15° winding angle. The resulting total thickness of both laminate structures corresponds to the research values from Table 2.

**Figure 5:**
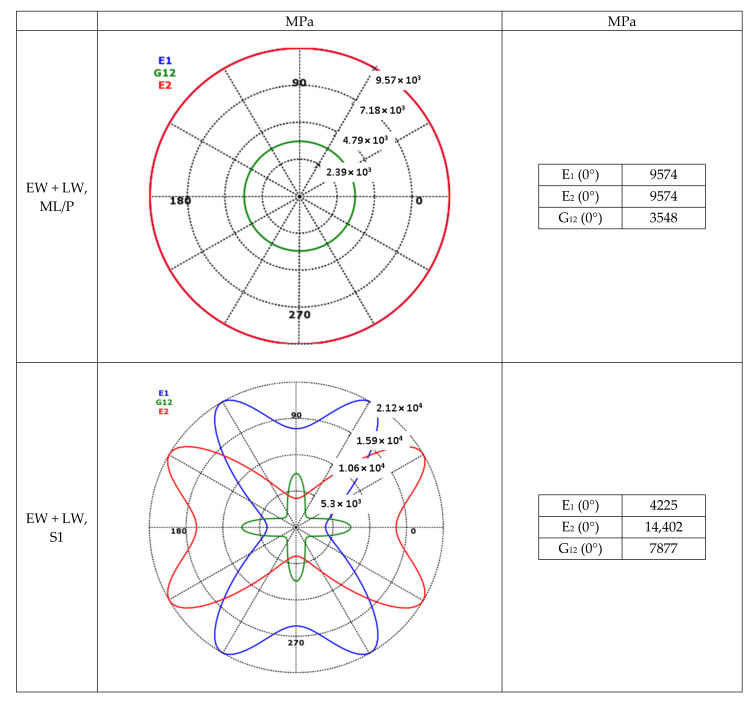

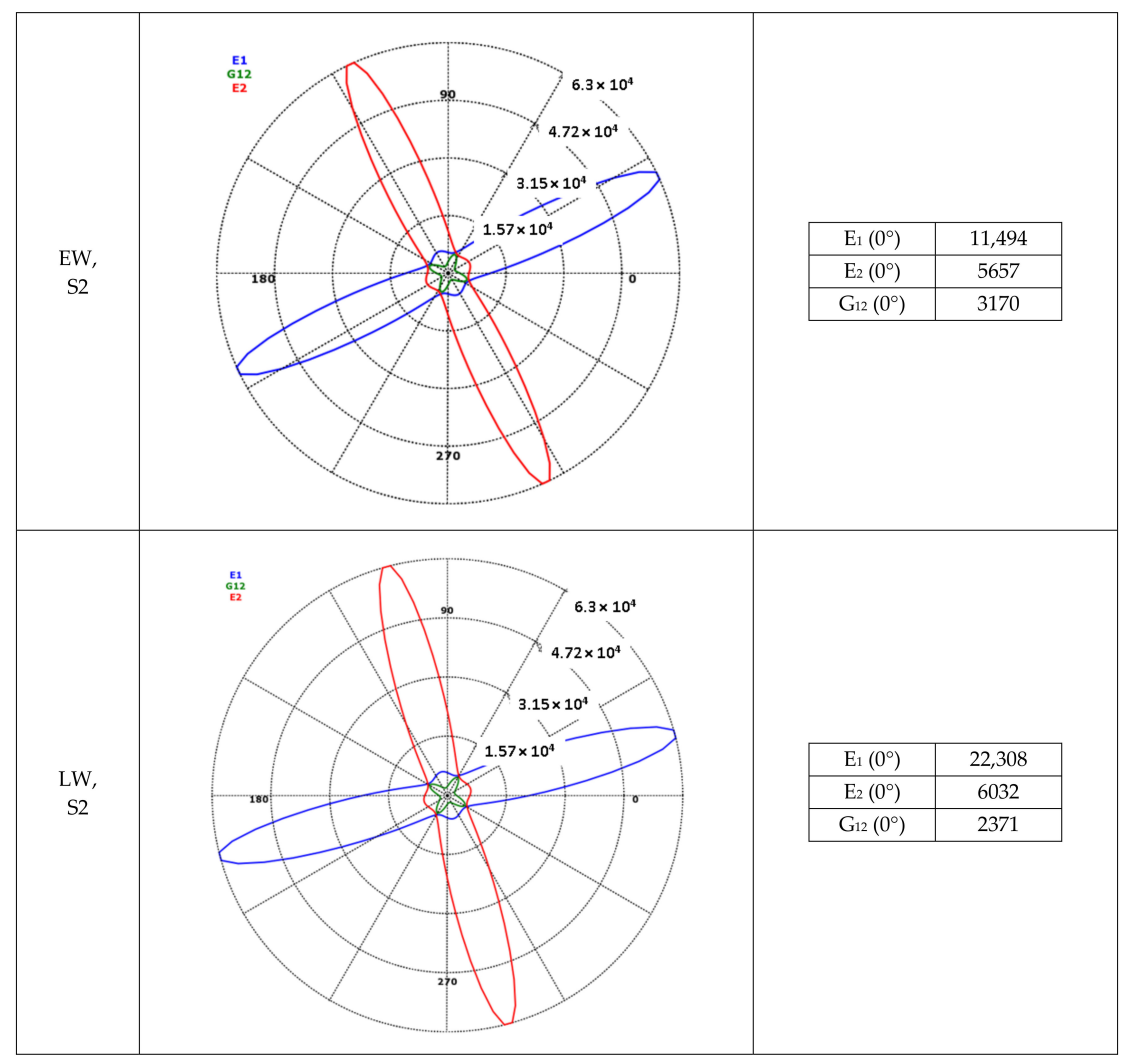

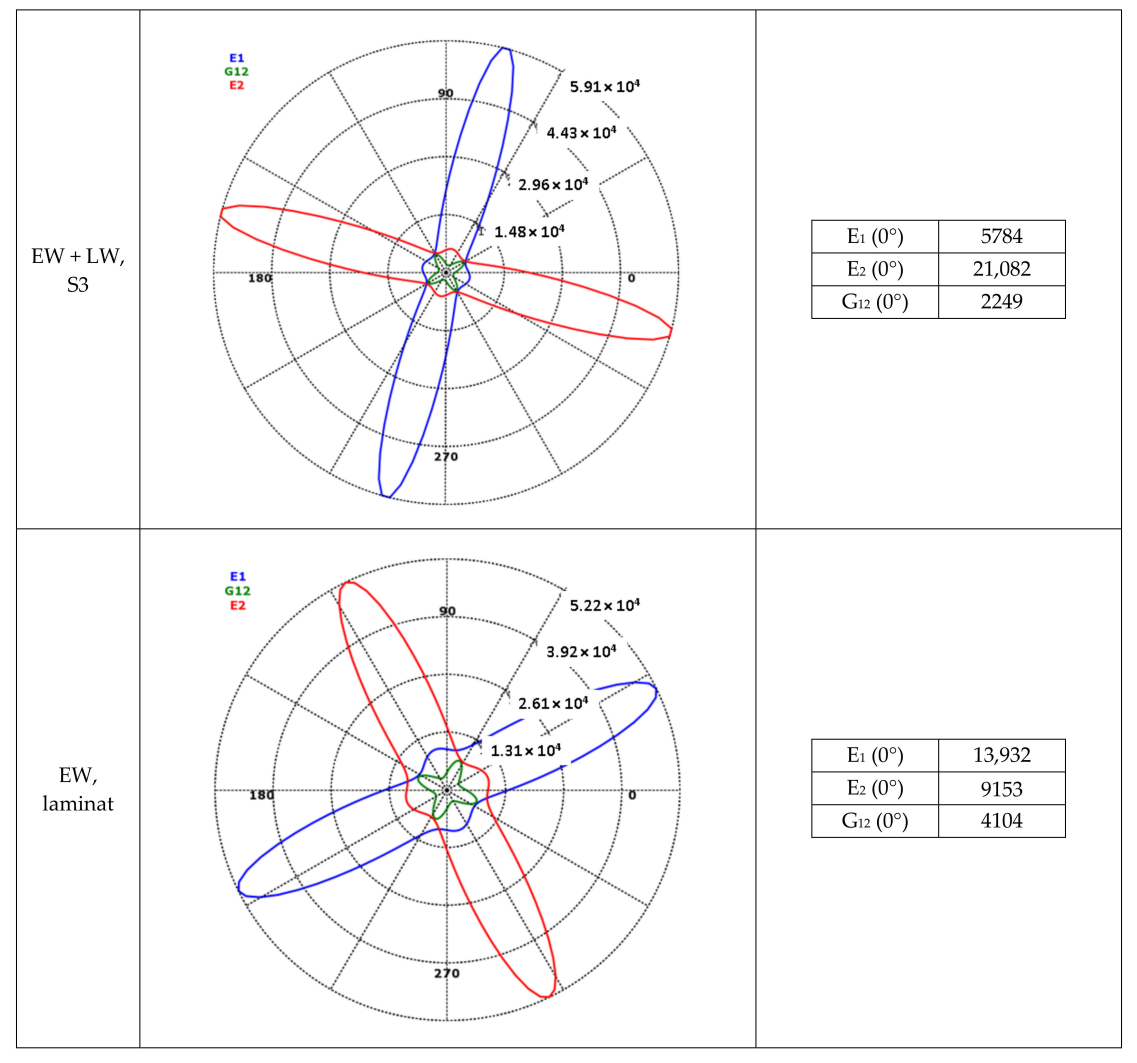

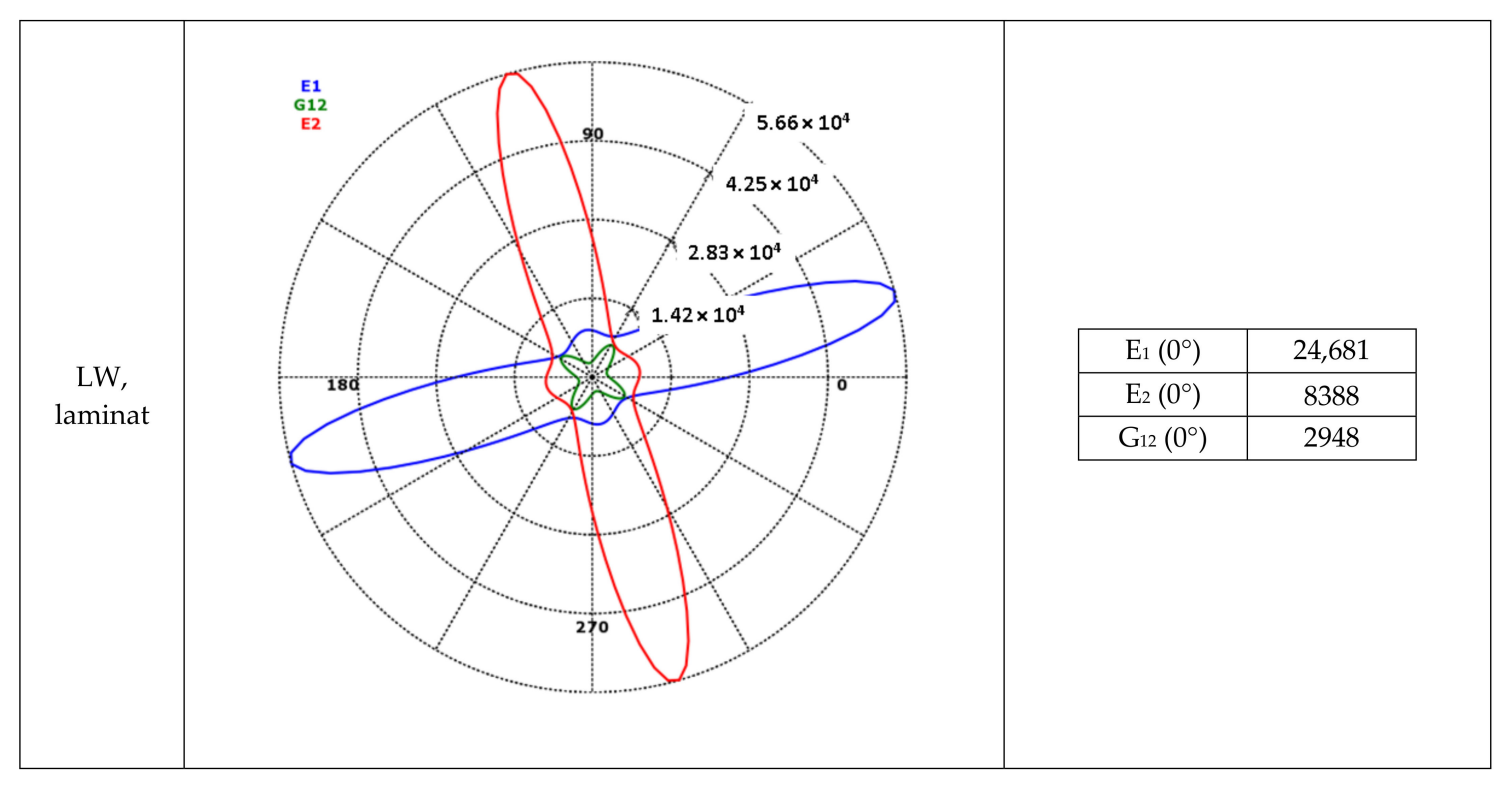
Calculated mechanical properties of cell wall layers of tracheids.

**Figure 6:**
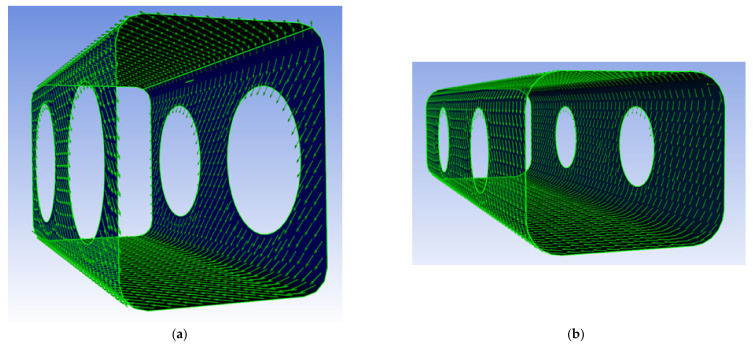
Modeling of fiber winding angle and layer thickness for EW (**a**) and LW tracheids (**b**) in Ansys ACP PrePost.

By combining the mechanical properties of the unidirectional cell wall single layers from Table 3 and the laminate structure from Table 4, the resulting mechanical properties of the individual cell wall single layers as well as the overall laminate structure were calculated, presented in polar diagram form (Figure 5) and used to replicate the cell wall in Software Ansys ACP PrePost, Version 2019 R1, ANSYS, Inc., Pennsylvania, US (Figure 6).

#### 2.1.3. Compression Behavior of Tracheids

**Figure 7:**
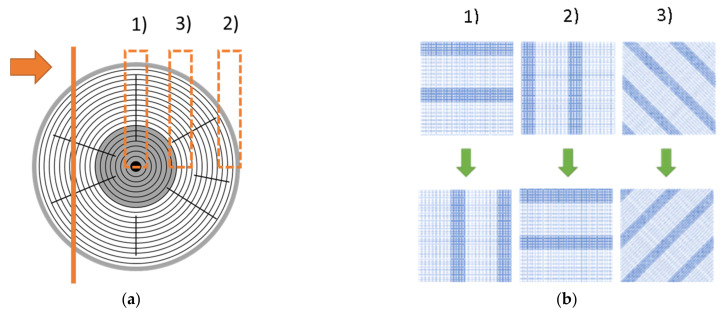
Schematic representation of the position of the cutting plane (**a**) and the resulting production-related variations of the EW and LW positions in height direction of the wood particle (**b**).

During compression perpendicular to the longitudinal axis of the wood particles, the EW and LW areas are subjected to tangential (Figure 7(b1)), radial (Figure 7(b2)) or mixed loads (Figure 7(b3)), depending on how the respective wood particle is cut out of the tree trunk (Figure 7a).

**Figure 8:**
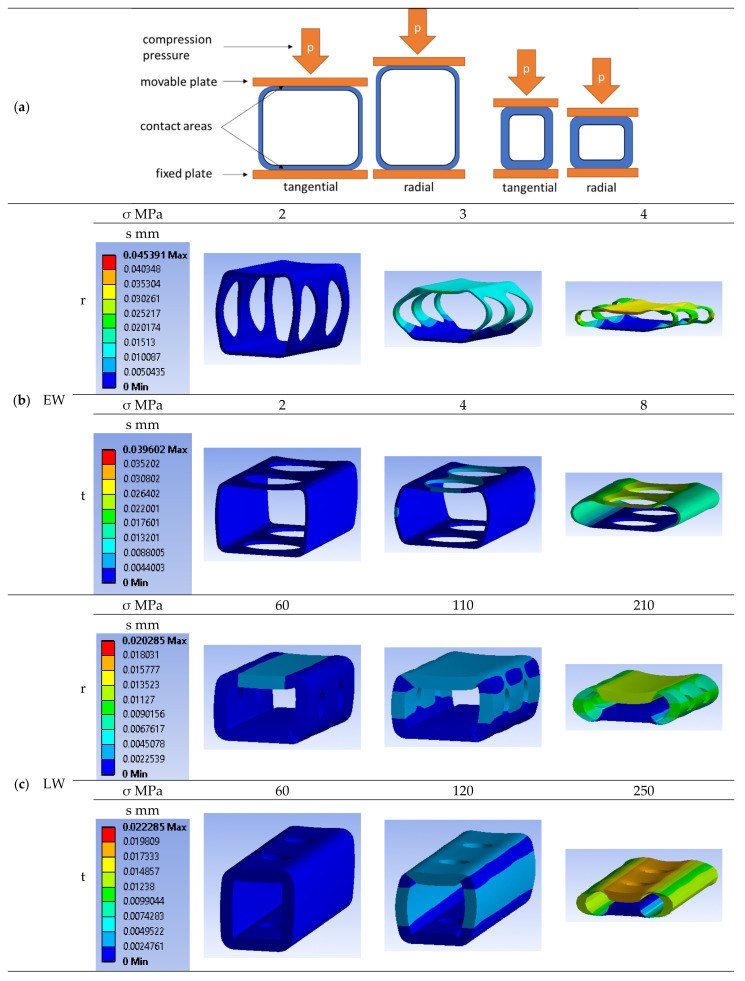
Selected boundary conditions (**a**) and simulation results of radial (r) and tangential (t) compression of EW (**b**) and LW (**c**) tracheids at 23 °C and 12% moisture content.

Therefore, it is necessary to simulate the tangential as well as the radial deformation behavior of the tracheids. Figure 8a shows the selected boundary conditions for the simulation using the FEM software Ansys Workbench Mechanical 2019 R1 (ANSYS, Inc., Canonsburg, PA, USA). The position of the lower side of the tracheid on the fixed plate was set by choosing the contact type “bonded” (separation in the surface normal direction and sliding in the surface tangential direction prohibited), and for the moving plate, the contact type “rough” (separation in the surface normal direction allowed but sliding in the surface tangential direction prohibited) was selected. The mesh settings were set to physics preset “mechanics”, element approach function “linear”, element size “0.003 mm”, adaptive size “no” and mesh defeaturing “yes”. Subsequently, the compression pressure on the movable plate was increased until the cell walls collapsed. Because the deformed and undeformed geometry differs significantly, the Ansys nonlinear adaptivity feature (NLAD) was activated to repair the mesh distortions that would trigger convergence issues. Figure 8b shows the deformation of an EW tracheid for radial as well as tangential compression at 23 °C and 12% moisture. In the radial direction, cell collapse starts at 2 MPa compression pressure and at 4 MPa, the tracheid is deformed by over 0.035 mm. In the tangential direction, cell collapse starts at 4 MPa compression pressure and at 8 MPa, the tracheid is deformed by over 0.023 mm. Figure 8c shows the deformation of an LW tracheid for radial as well as tangential compression at 23 °C and 12% moisture. In the radial direction, cell collapse starts at 110 MPa compression pressure and at 210 MPa, the tracheid is deformed by more than 0.013 mm. In the tangential direction, cell collapse starts at 120 MPa compression pressure and at 250 MPa, the tracheid is deformed by over 0.018 mm.

**Figure 9:**
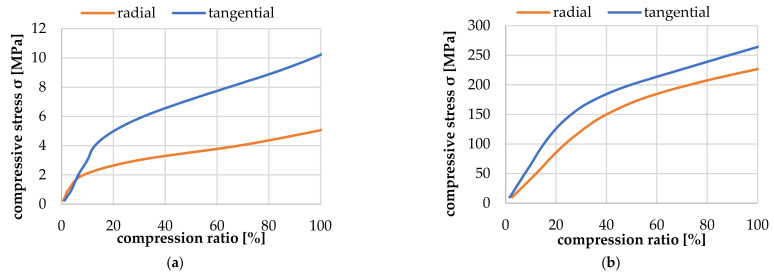
Simulation result for the compression behavior of an EW (**a**) and LW tracheid (**b**) at 23 °C and 12% moisture content.

Figure 9 shows the resulting correlation between compression pressure and compression ratio (ratio of deformation to total height minus twice the wall thickness) on the left side for an EW tracheid in radial and tangential directions. The stress plateau in the tangential direction is about 200% higher than in the radial direction, and the plateau already starts at a 10% compression ratio. On the right side of Figure 9, the resulting correlation between compression pressure and compression ratio for an LW tracheid in the radial and tangential direction is depicted. The stress plateau in the tangential direction is about 15% higher than in the radial direction, and the plateau already starts at a 30% compression ratio.

The simulation results show that at least 10 MPa must be applied for maximum compaction of the EW tracheids. The LW tracheids can withstand at least 100 MPa compression pressure and would deform elastically at this load by about 20%. Over 100 MPa compression pressure, the cell walls of LW tracheids start to collapse, and plastic deformation occurs, which must be avoided during the processing in order not to damage their fiber composite structure.

The following chapter presents measurement results for further validation of the simulation results mentioned above.

### 2.2. Materials

#### 2.2.1. Pine Wood Particles

**Figure 10:**
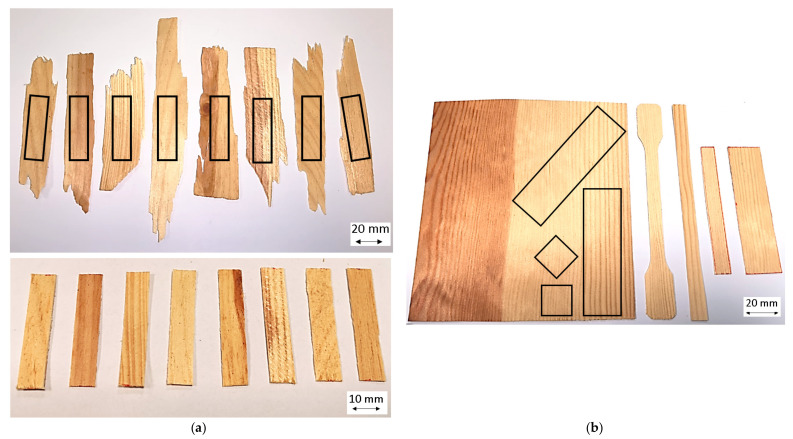
OSB strands and specimens taken therefrom using a punch (**a**) and pine veneer used and utilized specimen shapes (**b**).

The manufacturing-related high variation in growth characteristics and geometry (length 60 to 150 mm, width from 5 to 30 mm and thickness from 0.5 to 1 mm) of OSB strands limits the usable shape of the test specimens considerably (Figure 10a).

Furthermore, growth ring width, growth ring angle and the ratio of heartwood to sapwood vary considerably. This leads to a large deviation of the measurement results and makes it difficult to interpret the results and derive new knowledge to determine the optimum process parameters [49]. In order to avoid these measurement deviations, the tests were therefore carried out with veneer strips and squares, which show an even wood structure with parallel annual rings, allowing a defined separation between heart- and sapwood and samples, which can be cut out of the wood in a more defined manner (Figure 10b). For the mechanical tests, a 1 mm thick, lengthwise sliced pine veneer with an average density of 0.53 g/cm^3^ was purchased from Osenstätter GmbH Holz & Furnier, Schongau. The veneer is 16 cm wide with approx. 9 cm consisting of sapwood and approx. 7 cm of heartwood. It was exposed to laboratory climate at 23 °C and room humidity at 45% (measured with Trotec, BC06 hand-held measuring device) until a constant weight was reached before testing. The resulting water mass fraction was 8.3% for sapwood and 7.5% for heartwood (measured with Satorius, MA100 at 103 °C for 60 min.). To minimize the deviation of the measurement results, the samples were taken exclusively from the sapwood using a special glass fiber fabric scissor (R&G ERGO-STL).

#### 2.2.2. Matrix

A polypropylene powder (HC001A-B1, Borealis AG, Austria, Vienna) was used as a matrix material to produce WPC specimens. According to the supplier data sheet, the particle size ranges from 150 to 425 μm, the melting temperature is 162 °C, the density is 0.95 g/cm^3^ and the tensile modulus is 1350 MPa, respectively.

### 2.3. Methods and Experimental Setup

#### 2.3.1. Optical Examination of the Compression Mechanisms

To characterize interlaminar interactions and compression mechanisms, experimental preliminary tests on the compression behavior on the macro (wood particle) and meso-levels (growth rings and EW and LW tracheids) were conducted. At first, the pressure distribution within two wood particles in 0° and 0/90° arrangement was analyzed. For this purpose, a pressure-sensitive plastic measuring foil (KAGER-Prescale Medium, measuring range 10 to 50 MPa) was inserted between the wood particle layers before compressing them at 23 °C with 10 MPa without polymer matrix material.

Secondly, the compression of the cross-sectional area in the longitudinal axis of the wood particles was examined under a microscope (Carl Zeiss, Stemi 2000-C with AxioCam ICc1 digital image recorder) by pretreating the wood particles with 1000 grit sandpaper and gradually compressing them in a miniature vice.

Thirdly, using a VK-X250 laser scanning microscope from KEYENCE DEUTSCHLAND GmbH, Neu-Isenburg, a surface analysis was conducted to measure the indentation depth of the LW tracheids when compressing two particles as a function of their angular difference in the longitudinal direction.

#### 2.3.2. Tensile Tests of Veneer Strips

According to Hiller, no standardized method exists to determine the tensile properties of elongated wood particles [50]. Therefore, tensile tests based on DIN EN ISO 527-4 were conducted using at least five test specimens with a total length of 100 mm, a width of 25 mm and a thickness of 1 mm. The measuring length was 50 mm, and the testing speed was 1 mm/min. The tensile modulus of elasticity was calculated by determining the slope of the stress–strain curve between ε = 0.0005 and ε = 0.0025. This is permissible because most test standards for determining the modulus of elasticity of solid wood state that two value pairs out of the strain range before the proportionality limit are to be used.

#### 2.3.3. Compressive Tests of Veneer Strips and Squares Perpendicular to Longitudinal Axis

According to Hiller, no standardized methods to determine the compressive strength and modulus of veneer strips and squares perpendicular to their longitudinal axis exist [50]. Therefore, based on DIN EN 319, square-shaped test specimens were used with an edge length of 20 mm and thickness of 1 mm. The testing speed was 1 mm/min.

#### 2.3.4. Experimental Setup for Production of WPC Test Specimen

WPC test specimens were prepared with both 80 and 90% OSB pine strands by weight. For the 80% by weight samples, 200 g OSB pine strands (in six layers, i.e., 33.3 g per layer) and 50 g polypropylene powder (in seven layers, i.e., 7.1 g) were used. For the 90% by weight samples, 200 g OSB pine strands (in six layers, i.e., 33.3 g per layer) and 22 g polypropylene powder (in seven layers, i.e., 3.2 g) were used.

**Figure 11:**
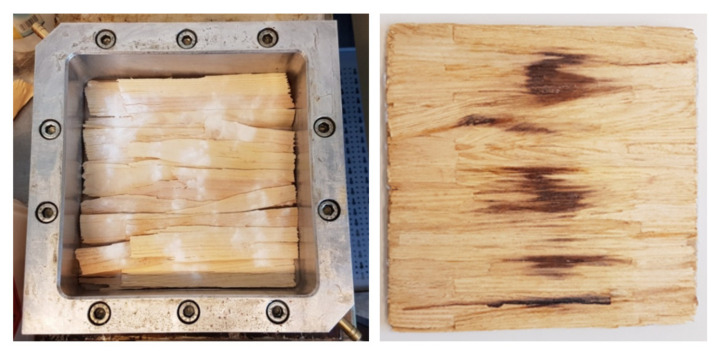
Manual production of WPC specimens consisting of OSB strands and thermoplastic powder.

The amount of powder was weighed individually for each layer and applied manually using a powder spreader (Figure 11).

#### 2.3.5. Tensile Tests WPC

**Figure 12:**
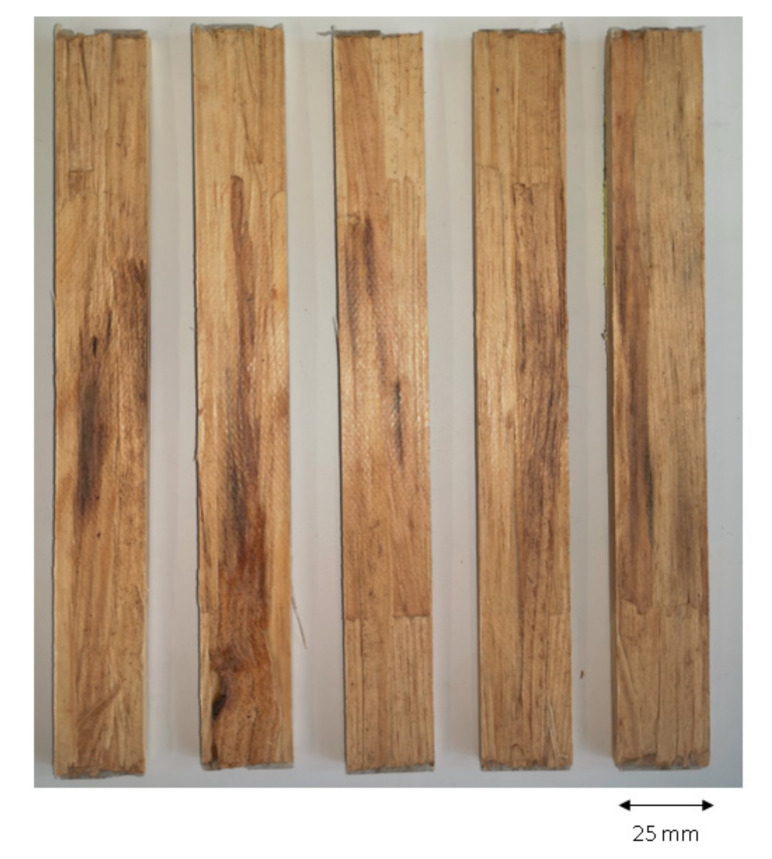
Manufactured WPC specimens for tensile testing.

To determine the tensile properties of the WPC, test specimens were used based on DIN EN ISO 527-4 with a total length of 200 mm, width of 25 mm and thickness of 6 mm (Figure 12).

The measuring length was 120 mm, and the testing speed was 1 mm/min. The tensile modulus of elasticity was calculated by determining the slope of the stress–strain curve between ε = 0.0005 and ε = 0.0025.

## 3. Results

### 3.1. Optical Examination of the Compression Mechanisms

**Figure 13:**
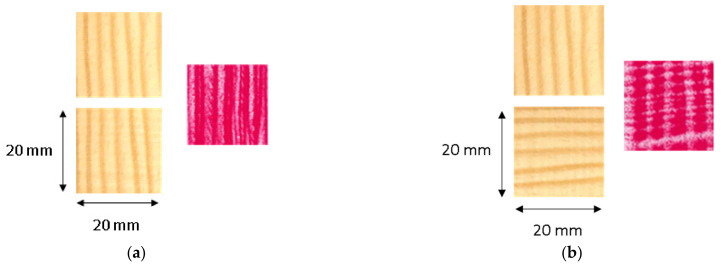
Formation of characteristic patterns when compressing pine veneer rectangles with a 0° (**a**) and 0/90° layer angle difference (**b**).

**Figure 14:**
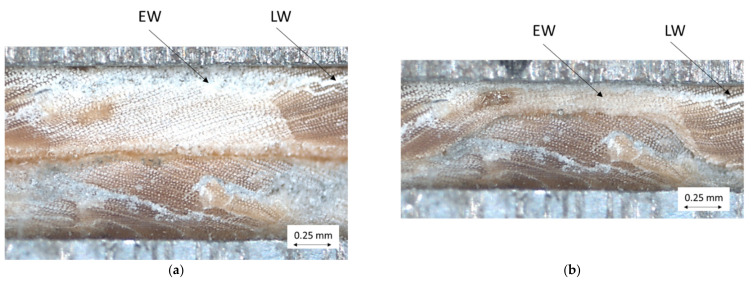
Compression of two layered wood particles with stacked LW and EW areas—initial state (**a**), deformed state (**b**).

**Figure 15:**
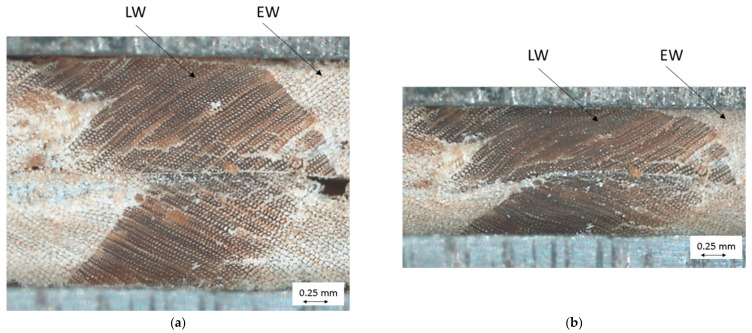
Compression of two layered wood particles with two stacked LW areas—initial state (**a**), deformed state (**b**).

Figure 13 shows a very even pressure distribution in the 0° particle arrangement because the areas of the more stable LW tracheids are pressed into the areas of the EW tracheids and deform them (Figure 14). At 0/90° arrangement, Figure 13 shows a grid-like pressure distribution due to the crossing points of the mechanically more stable areas of LW tracheids, which are laterally deformed in the direction of the sample width (Figure 15).

**Figure 16:**
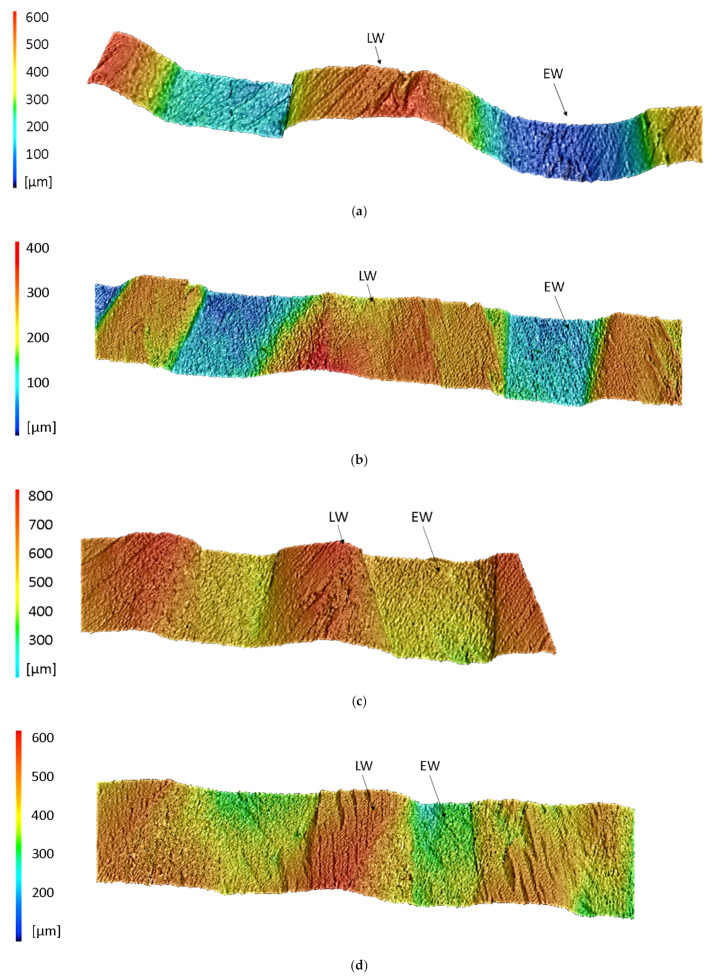
Sections of surface profiles of wood particles that were compacted with 10 MPa with an angular difference in the longitudinal direction of 0° (**a**), 5° (**b**), 10° (**c**) and 15° (**d**) for 10 min. at 23 °C.

Figure 16 shows sections of the surface profiles of two wood particles that were compacted with 10 MPa for 10 min. at 23 °C. As presumed above, the degree of compaction of the EW areas decreases with increasing angle differences. The measured indentation depth of the LW areas at 0° was about 0.49 mm (Figure 16a), at 5° about 0.44 mm (Figure 16b), at 10° about 0.28 mm (Figure 16c) and at 15° about 0.26 mm (Figure 16d).

### 3.2. Tensile Tests of Pine Sapwood Veneer Strips

#### 3.2.1. Material-Related Influencing Factors

**Figure 17:**
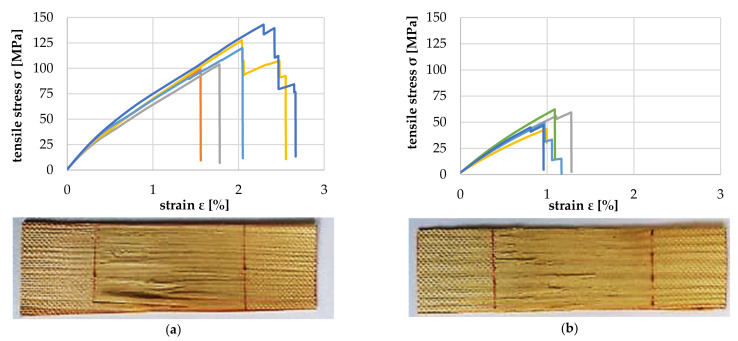
Tensile properties for a growth ring width of 1 mm (**a**) and 2 mm (**b**).

Figure 17 shows the comparison of the tensile properties of the above-mentioned veneer strips with 1 mm and 2 mm growth ring width (GRW). As expected, due to the higher proportion of LW, the tensile strength of the veneer with 1 mm GRW was twice as high as that of the veneer with 2 mm. Additionally, the elongation at the break of the veneer with 1 mm GRW was about 15% higher and in the range of approx. 1.6% (orange curve) to 2.3% (dark blue curve). The average longitudinal modulus of elasticity $\bar{E}$ for GRW 1 mm was 8819.6 (+713.2/−629.2) MPa, and for GRW 2 mm, 5841.6 (+738.8/−886.8) MPa.

**Figure 18:**
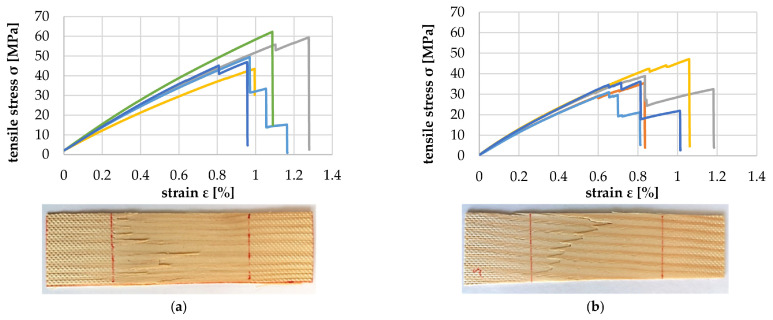

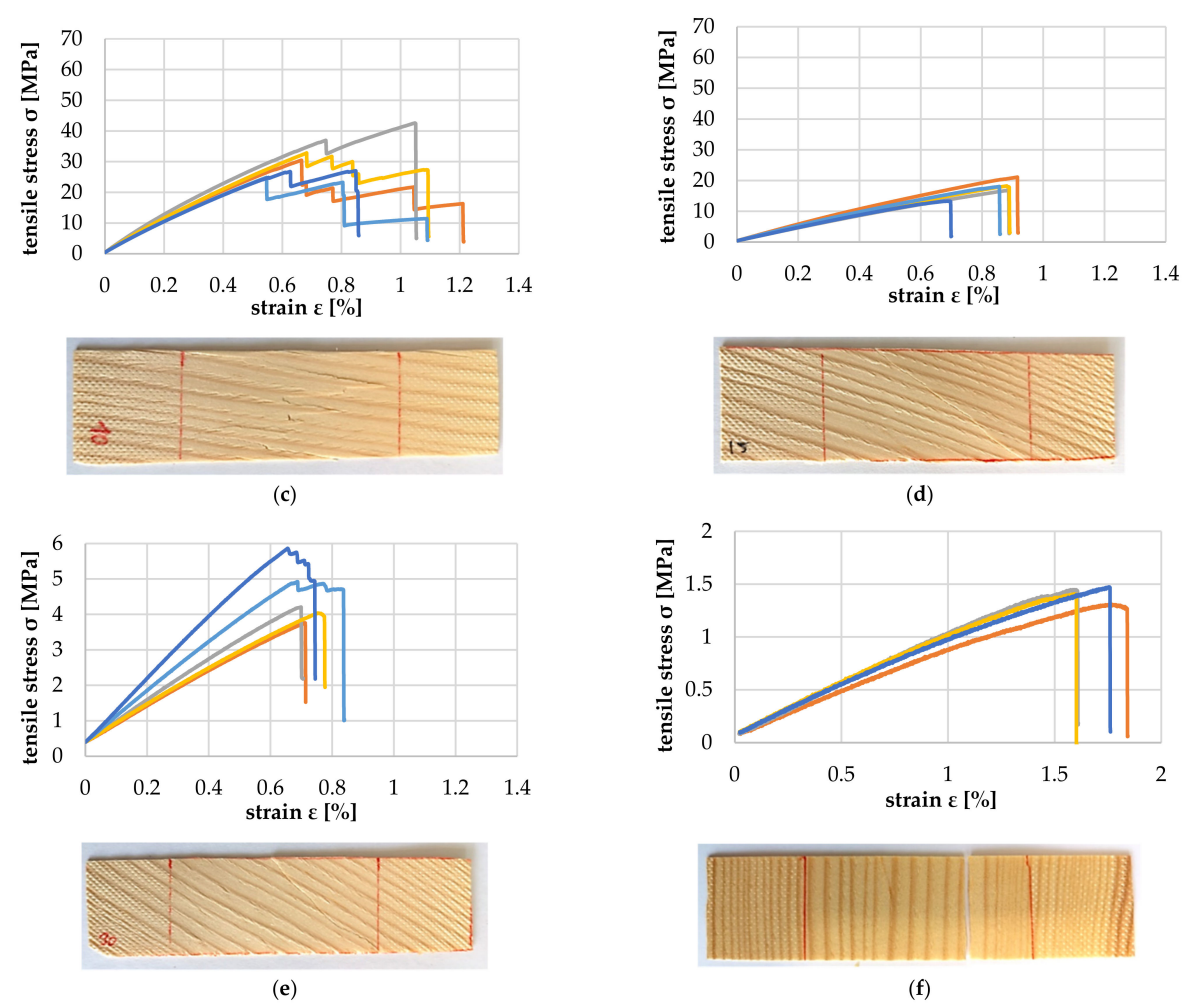
Tensile test at 0° (**a**), 5° (**b**), 10° (**c**), 15° (**d**), 30° (**e**) and 90° (**f**) deviation between particle and tracheid longitudinal axes.

**Figure 19:**
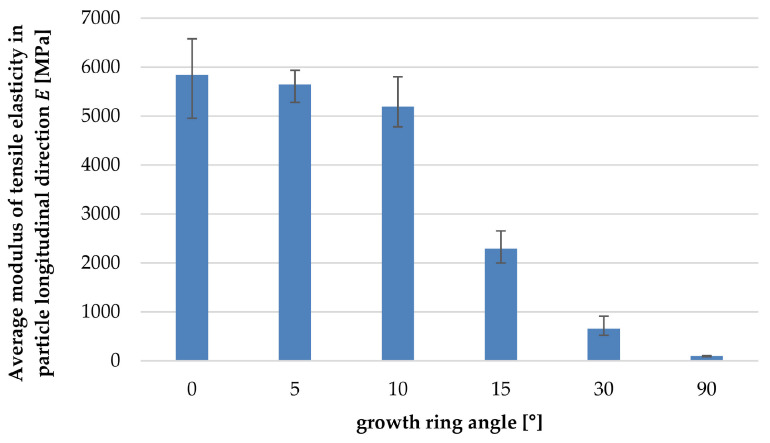
Average modulus of tensile elasticity in particle longitudinal direction as a function of growth ring angle.

Figure 18a–f show the tensile properties of the particles with the increasing deviation between annual ring angle and load direction. It is shown that from a deviation of 10°, there was a significant reduction in the tolerable tensile load and modulus of tensile elasticity (Figure 19). The failure mechanism changes from longitudinal tearing of LW tracheids, which can be seen in the stepped progressions of the stress–strain curves, to the formation of transverse cracks mainly in the EW areas, which can be seen in the abrupt drop in the stress–strain curve.

#### 3.2.2. Process-Related Influencing Factors

**Figure 20:**
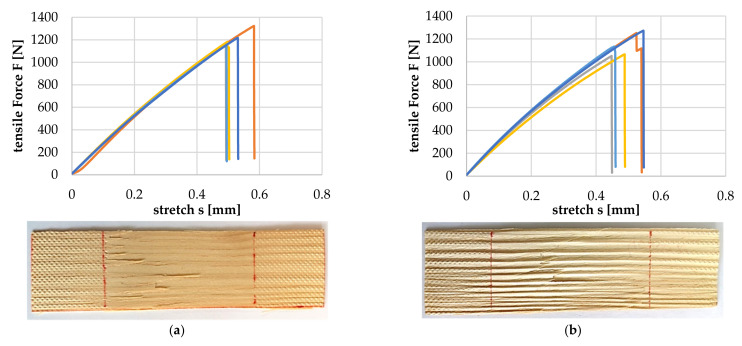
Comparison of tensile test of uncompressed (**a**) and compressed wood particles (**b**).

To examine whether the LW tracheids are damaged during compaction and their tensile strength is reduced as a result, two wood particles (GRW 2 mm) were compressed with a pressure of 10 MPa at 23 °C for 10 min., and then every single particle was evaluated via tensile testing (Figure 20).

The average tolerable tensile force for the uncompressed wood particles was 1204.4 (+116.4/−53.4) N, and for the compressed wood particles, it was 1153.6 (+117.4/−102.6) N. The deviation of approx. 4% was within the known scatter range of the wood used. This suggests that no damage to the LW tracheids took place.

**Figure 21:**
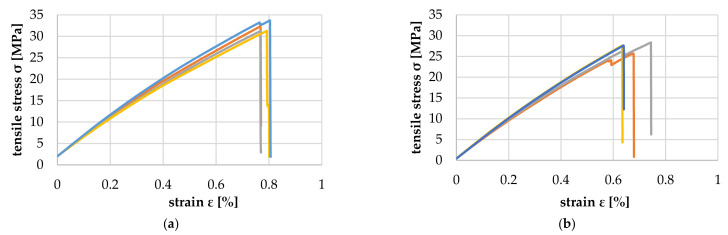
Tensile test at 0° deviation between particle and tracheid longitudinal axes tempered at 120 (**a**) and 170 °C (**b**) for 20 min.

Figure 21 shows the tensile test for wood particles that were tempered at 120 and 170 °C, respectively, for 20 min before testing. The tensile modulus of elasticity shows a deviation of 2% and can be considered similar for both specimens (4597.6 (+530.0/−339.2) MPa for the 120 °C specimens and 4510.1 (+238.8/−205.7) MPa for the 170 °C specimens), but the tensile strength and elongation at break were about 15% lower for the 170 °C specimens.

**Figure 22:**
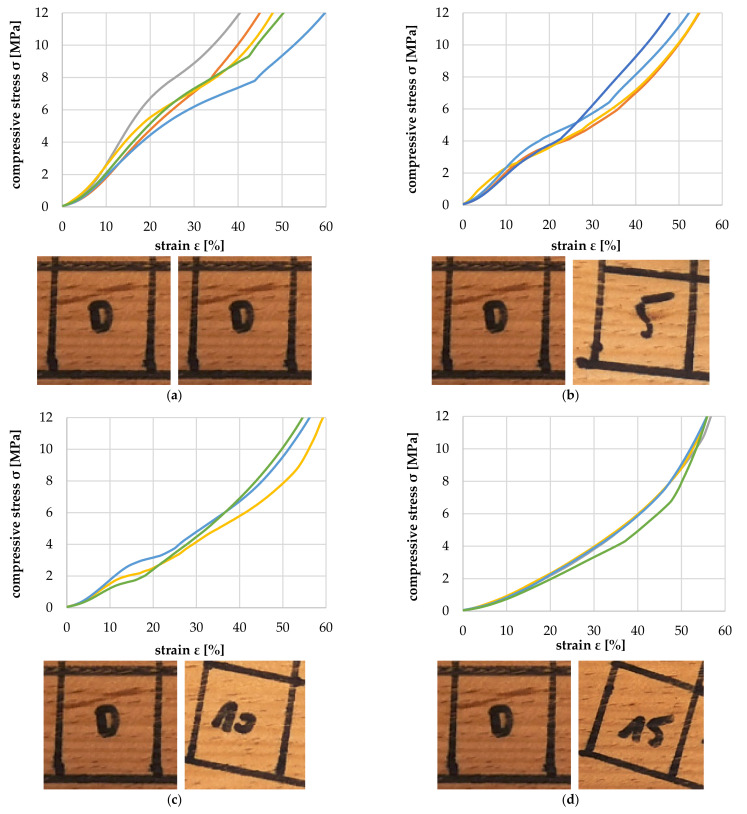
Compressive test with 0° (**a**), 5° (**b**), 10° (**c**) and 15° (**d**) deviation in the longitudinal direction.

Figure 22a–d show the stress–strain curves for the compression of two layers (each 1 mm thick) of veneer squares (GRW 2 mm) perpendicular to their longitudinal axis with 0°, 5°, 10° and 15° angular differences in the longitudinal direction. For the 0°, 5° and 10° tests, a plateau in the stress–strain curves can be seen in the range of 10 to 30% strain. The area under the plateau indicates the work that is required to deform the EW tracheid areas (similar to the compression of foam materials and automotive crash absorbers).

The measurements show that by compressing two wood particles with no angular deviation in the longitudinal direction, the LW areas are pressed into the EW areas, and the EW tracheids are, corresponding to the simulation results shown in Figure 9 and measurement results shown in Figure 16, compacted in the highest possible manner at 10 MPa (Figure 22a). If there is an angular deviation in the longitudinal direction greater than 10°, the LW areas form a lattice-like structure which results in less compaction of the EW tracheids and more deformation of the LW areas.

### 3.3. Tensile Tests of WPC with Polypropylene Matrix

**Figure 23:**
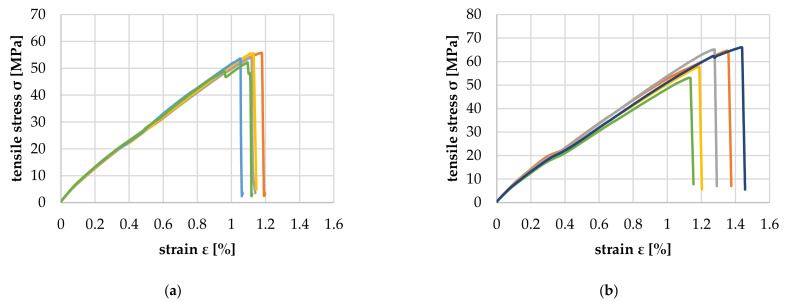
Parallel tensile properties of PP-based WPC with 80 (**a**) and 90 wt% (**b**) wood content.

Figure 23 shows the comparison of the parallel tensile properties of PP-based WPC with 80 and 90 wt% wood content. The measured tensile modulus of elasticity is similar for both WPC with 5881.1 (+459.9/−177.0) MPa for 80 and 5865.5 (+560.9/−306.9) MPa for 90 wt%, respectively. However, the tensile strength and the elongation at break are about 10% lower for the 80 wt% specimens.

**Figure 24:**
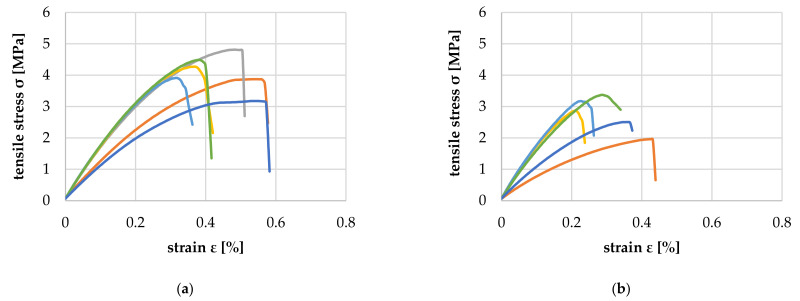
Transverse tensile properties of PP-based WPC with 80 (**a**) and 90 wt% (**b**) wood content.

Figure 24 shows the comparison of the transverse tensile properties of PP-based WPC with 80 and 90 wt% wood content. The tensile modulus of elasticity achieved was 1180.1 (+158.6/−334.7) MPa for 80 wt%, about 40% higher than the 845.6 (+305.9/−304.8) MPa for 90 wt% specimens because the proportion of matrix that determines transverse strength was only 10 wt%. As expected, the tensile strength and elongation at break were about 40% lower for the 90 wt% specimens.

### 3.4. Analytical Estimation of Achievable Mechanical Properties of WPC

A first simplified approach to estimate the achievable tensile elasticity parallel to the particle direction ($E_{1}$) for a unidirectional reinforced pine wood strand polypropylene composite with 90 wt% wood content was conducted by applying the rule of mixture (1) known from continuous fiber composite technology as well as an equation for converting mass to volume fraction (2).

(1) $$E_{1}=E_{p,1}\times \varphi +E_{M}\times \left(1-\varphi \right)$$

(2) $$\varphi =\frac{\psi \times \rho_{M}}{\psi \times \rho_{M}+\left(1-\psi \right)\times \rho_{p}}$$

$E_{p,1}$ is the modulus of elasticity of the pine wood particle in its longitudinal direction. $E_{M}$ is the modulus of elasticity of the polypropylene matrix (1350 MPa). φ is the particle volume content and ψ is the particle mass content. $\rho_{p}$ is the particle density and $\rho_{M}$ the matrix density (0.95 g/cm^3^). To estimate $E_{1}$ the material parameters as well as the process conditions must be taken into account. By assuming that the average growth ring width is 2 mm (Figure 17b), the average growth ring angle inclination in the longitudinal axis is 0° (Figure 18a), the mold temperature was 170 °C (Figure 21) and the compression pressure was 10 MPa (Figure 22a) $E_{1}$ can be estimated as follows (3)–(6):

(3) $$E_{p,1}{}^{*}=\frac{E_{p,1}\left(T=170\;{}^{\circ}C\right)}{1-\varepsilon \left(p=10\;\mathrm{MPa}\right)}=7516\;\mathrm{MPa},$$

(4) $$\rho_{p}{}^{*}=\frac{\rho_{p}\left(p=0\;\mathrm{MPa}\right)}{1-\varepsilon \left(p=10\;\mathrm{MPa}\right)}=0.875\;g/{\mathrm{cm}}^{3},$$

(5) φ=91 vol%

(6) $$E_{1}=6961\;\mathrm{MPa}.$$

This results in an overestimation of $E_{1}$ of about 19% (Figure 23b), which is understandable considering that it is a discontinuously reinforced composite with interactions between the reinforcing elements. Further research is planned to increase the accuracy of the estimation model.

## 4. Discussion

With the help of the simulations and experiments, it was shown that through targeted layering and the compression of softwood particles, it is possible to significantly increase their mechanical properties. This is enabled by pressing areas with LW tracheids into areas with EW tracheids, thereby strongly compressing the areas of EW tracheids and increasing the total volume fraction of LW tracheids. Because the mechanical properties of the tracheid cell wall are highly dependent on temperature and moisture content, the compressive strength of the tracheids perpendicular to their longitudinal axis must therefore be estimated for the prevailing process conditions [22,23]. The simulation model presented in this work can be used for this purpose. According to the findings of this work, at 23 °C and 12% moisture content, at least 10 MPa must be applied for maximum compaction of the EW tracheids. According to research, the measured compressive strength of solid pine wood is 4–14 MPa in the radial and 8 MPa in the tangential direction [11,51]. The presented modeling approach thus maps the material behavior determined by measurement with sufficient accuracy. The remaining discrepancies can be explained by growth-related fluctuations in dimensions, wall thickness and cellulose fiber content of the tracheids, as well as the mutual support of the cell walls in the tracheid network and the multiaxial load conditions in the solid wood [10]. This behavior could be represented realistically by arranging and compressing the developed EW and LW tracheid models, such as a larger network similar to reference [27]. The LW tracheids can withstand at least 100 MPa compression pressure and would deform elastically at this load by about 20%. Over 100 MPa compression pressure, the cell walls of LW tracheids start to collapse, and plastic deformation occurs, which must be avoided during the processing to avoid damaging their strength-enhancing fiber composite structure, primarily the orientation and degree of undulation of the cellulosic fibers of the S2 layer. For wood–polymer composites with more complex laminate structures with load-adapted particle orientation, a lattice-like structure of areas of LW tracheids will be generated, which requires further research to investigate the vertical pressure distribution in the cell network while avoiding local pressure “hot spots”.

## 5. Conclusions

An RVE- and FEM-based model was developed to simulate the radial and tangential compression behavior of softwood tracheids at 12% wood moisture content and 23 °C temperature using pine as an example. This model can be adapted for more complex load types (e.g., mixed shear compression) and other wood species by adjusting the mechanical properties of the base materials of the cell wall single layers (cellulose, hemicellulose, lignin), the dimensions and the structure of the vessel elements. With the help of this model, the exact compression pressure can be determined at which the EW tracheids are compressed to the maximum, and the LW tracheids are deformed purely elastically so that their fiber composite structure remains undamaged. This type of targeted compaction of softwood, in combination with the load-oriented positioning of the wood particles, enables the production of innovative wood–plastic composite components, allowing new application possibilities for softwood in the automotive, aircraft and mechanical engineering industries [52].

## Figures and Tables

**Table 1:** Mechanical properties of the base materials of the cell wall single layers.

	E_1_	E_2_	ν_12_	ν_23_	G_12_
	MPa	MPa	-	-	MPa
cellulose	138,000	17,700	0.005	0.52	4500
hemicellulose	8000	3400	0.3	0.4	1000
lignin	3100	3100	0.33	0.33	1200

**Table 2:** Structural composition of EW and LW tracheids.

	EW		LW		Dry Mass Fraction		
	Thickness	Microfibril Angle	Thickness	Microfibril Angle	C	HC	L
	µm	°	µm	°	wt%	wt%	wt%
ML/P	0.11	random	0.1	random	15	32	53
S1	0.25	±50 to 70	0.34	±50 to 70	28	31	41
S2	1.54	10 to 40	3.84	0 to 30	50	31	19
S3	0.06	60 to 90	0.09	60 to 90	48	36	16
cell wall	1.96		4.37				

**Table 3:** Mechanical properties of the unidirectional cell wall single layers determined by RVE modeling for 12% moisture and 23 °C temperature (hemicellulose—blue, cellulose—red, lignin—green).

		ML/P	S1	S2	S3
RVE Mesh		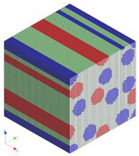	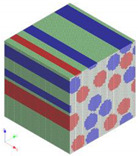	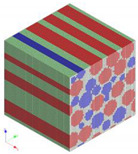	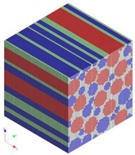
density	g/cm^3^	1.42	1.44	1.47	1.48
C	wt%	13	25	44	42
HC	wt%	28	27	27	32
L	wt%	47	36	17	14
water	wt%	12	12	12	12
E_1_	MPa	21,562	38,193	63,550	59,636
E_2_	MPa	4019	4790	6435	6187
G_12_	MPa	1308	1541	1997	1892
ν_12_	-	0.38	0.40	0.44	0.44

**Table 4:** Number of layers, layer thickness and winding angle used for tracheid modeling.

	EW		LW	
	Winding Angle	Layer Thickness	Winding Angle	Layer Thickness
	°	mm	°	mm
ML/P	0	0.0000275	0	0.000025
	45	0.0000275	45	0.000025
	−45	0.0000275	−45	0.000025
	90	0.0000275	90	0.000025
S1	60	0.000125	60	0.00017
	−60	0.000125	−60	0.00017
S2	25	0.00154	15	0.0025
S3	75	0.00006	75	0.00009
total		0.00196		0.00437

## Data Availability

Not applicable.
